# Footprint of Positive Selection in *Treponema pallidum* subsp. *pallidum* Genome Sequences Suggests Adaptive Microevolution of the Syphilis Pathogen

**DOI:** 10.1371/journal.pntd.0001698

**Published:** 2012-06-12

**Authors:** Lorenzo Giacani, Sujay Chattopadhyay, Arturo Centurion-Lara, Brendan M. Jeffrey, Hoavan T. Le, Barbara J. Molini, Sheila A. Lukehart, Evgeni V. Sokurenko, Daniel D. Rockey

**Affiliations:** 1 Department of Medicine, University of Washington, Seattle, Washington, United States of America; 2 Department of Microbiology, University of Washington, Seattle, Washington, United States of America; 3 Department of Biomedical Sciences, Oregon State University, Corvallis, Oregon, United States of America; 4 Department of Global Health, University of Washington, Seattle, Washington, United States of America; Institut Pasteur, France

## Abstract

In the rabbit model of syphilis, infection phenotypes associated with the Nichols and Chicago strains of *Treponema pallidum* (*T. pallidum*), though similar, are not identical. Between these strains, significant differences are found in expression of, and antibody responses to some candidate virulence factors, suggesting the existence of functional genetic differences between isolates. The Chicago strain genome was therefore sequenced and compared to the Nichols genome, available since 1998. Initial comparative analysis suggested the presence of 44 single nucleotide polymorphisms (SNPs), 103 small (≤3 nucleotides) indels, and 1 large (1204 bp) insertion in the Chicago genome with respect to the Nichols genome. To confirm the above findings, Sanger sequencing was performed on most loci carrying differences using DNA from Chicago and the Nichols strain used in the original *T. pallidum* genome project. A majority of the previously identified differences were found to be due to errors in the published Nichols genome, while the accuracy of the Chicago genome was confirmed. However, 20 SNPs were confirmed between the two genomes, and 16 (80.0%) were found in coding regions, with all being of non-synonymous nature, strongly indicating action of positive selection. Sequencing of 16 genomic loci harboring SNPs in 12 additional *T. pallidum* strains, (SS14, Bal 3, Bal 7, Bal 9, Sea 81-3, Sea 81-8, Sea 86-1, Sea 87-1, Mexico A, UW231B, UW236B, and UW249C), was used to identify “Chicago-“ or “Nichols -specific” differences. All but one of the 16 SNPs were “Nichols-specific”, with Chicago having identical sequences at these positions to almost all of the additional strains examined. These mutations could reflect differential adaptation of the Nichols strain to the rabbit host or pathoadaptive mutations acquired during human infection. Our findings indicate that SNPs among *T. pallidum* strains emerge under positive selection and, therefore, are likely to be functional in nature.

## Author Summary

During infection, the agent of syphilis, *Treponema pallidum* subsp. *pallidum* (*T. pallidum*), successfully evades the host immune defenses and establishes a persistent infection that can cause blindness, paralysis, or even death in some individuals that progress to the tertiary stage of the disease. The study of the Nichols strain of *T. pallidum*, isolated over a century ago and continually propagated in rabbits, has been paramount to deepen our knowledge on the biology of the agent of syphilis and the pathogenesis of this complex disease. Nonetheless, when the more recent Chicago isolate of *T. pallidum* is compared to the Nichols strain, significant differences in gene expression, gene conversion rates, and antibody responses against virulence factor candidates are detected during experimental infection. To investigate whether differences at the genomic level between Nichols and Chicago might explain such phenotypic differences, we sequenced the Chicago strain genome and compared it to the previously sequenced *T. pallidum* Nichols strain. Our findings indicate that the genomic differences between these *T. pallidum* strains emerge under positive selection, and are likely to be functional in nature, thereby being involved in shaping the phenotypic diversity between the Chicago and Nichols strains.

## Introduction

Syphilis continues to be a common and serious disease, affecting at least 25 million persons worldwide [1]. It is a recognized cofactor in the transmission and acquisition of HIV [2], [3], and is a major cause of stillbirth and perinatal morbidity particularly in the developing world [4], [5]. The peculiar biology of the causative agent of syphilis, *Treponema pallidum* subspecies *pallidum* (*T. pallidum*), along with the inability to grow this pathogen continually *in vitro*, has hindered progress in understanding the pathogenesis of this disease. Syphilis research however, greatly benefited from the elucidation of the *T. pallidum* Nichols strain genome sequence [6]. This 1.138 Mb genome is among the smallest characterized in prokaryotes. The lack of genes encoding for several metabolic pathways (i.e. Krebs' cycle, glyoxylate shunt, amino acid and fatty acid synthesis, etc.), restriction-modification enzymes, transposons, or prophages [6], strongly suggests that *T. pallidum*'s evolution as a human pathogen exploited progressive genome reduction and loss of those functions now provided by the host.

Since its isolation in 1912 from the cerebrospinal fluid (CSF) of a patient with secondary syphilis [7], the Nichols strain of *T. pallidum* has been continually propagated in rabbits, and has become the reference strain in experimental syphilis. Thus, it was the obvious choice for the original *T. pallidum* genome project. The Chicago strain of *T. pallidum*, isolated in 1951 by Turner and Rodriguez from a primary chancre [8] and far less extensively propagated in rabbits, has become increasingly important in the study of the pathogenesis of syphilis. Despite the fact that Nichols and Chicago belong to the same *T. pallidum* molecular strain type (14a/a [9]), suggesting an elevated degree of genetic similarity, several phenotypic and genotypic differences have been highlighted between these strains during experimental infection. Important differences between the two strains were described regarding gene expression of candidate virulence factors [10], [11], as well as antibody and cellular responses against Nichols and Chicago antigens during experimental infection [10], [11]. An example of the above differences involves the 12-membered *tpr* (*T. pallidum* repeat) gene family [12]. The *tpr* genes and the antigens they encode have been the focus of intense research by our group, leading to the characterization of the immune response against these antigens during experimental syphilis [10], [11], and their potential as protective antigens [12]–[16]. The study of transcriptional patterns of these genes and the mechanisms that control expression of several *tpr* genes resulted in the identification of phase variation as a mechanism for controlling expression of at least five *tpr* genes [11], [17]. Another member of the *tpr* gene family, *tprK*, undergoes extensive sequence variation mediated by gene conversion during infection, resulting in changes in seven discrete variable (V) regions in the *tprK* ORF [18]. Chicago has been shown to diversify the sequence of *tprK* at a significantly higher baseline rate than Nichols, before onset of detectable specific immunity, during intratesticular (IT) passages and intradermal (ID) infections. The *tprK* gene in the Nichols strain remains virtually clonal, varying its sequence only after onset of an adaptive immune response against the initial TprK antigen [19], while variants arise throughout infection with the Chicago strain. In the presence of an adaptive immune response against the TprK antigen, the difference in accumulation of variants in Chicago is even more striking.

To investigate whether genomic differences could explain the biological differences between Nichols and Chicago, the genome of the Chicago strain was elucidated using next-generation Illumina sequencing, annotated, and compared to the published Nichols genome. Genomic differences were confirmed by dideoxy-terminator (DT) sequencing using template DNA from Chicago as well as the Nichols strain (Houston) used for the original *T. pallidum* genome project. All coding sequences carrying SNPs, as well as approximately one third of the loci carrying small indels, were amplified and sequenced, revealing a strikingly high frequency of sequencing errors in the available Nichols genome. Nonetheless, comparison of 16 Nichols and Chicago polymorphic loci with the corresponding genomic regions of 12 more recently isolated *T. pallidum* strains (SS14, Bal 3, Bal 7, Bal 9, Sea 81-3, Sea 81-8, Sea 86-1, Sea 87-1, Mexico A, UW231B, UW236B, and UW249C) suggested that the genetic differences between Chicago and Nichols were acquired under the action of positive selection, and allowed us to speculate on the pathoadaptive nature of these changes in these *T. pallidum* strains.

## Methods

### Ethics statement

No investigations were undertaken using humans/human samples in this study. New Zealand white rabbits were used for *T. pallidum* propagation. Animal care was provided in accordance with the procedures outlined in the Guide for the Care and Use of Laboratory Animals under protocols approved by the University of Washington Institutional Animal Care and Use Committee (IACUC).

### *T. pallidum* strain propagation and harvest, bacterial cell purification, and DNA isolation

The Chicago strain of *T. pallidum* subsp. *pallidum*, initially supplied by Dr. Paul Hardy and Ellen Nell (Johns Hopkins University, Baltimore, MD), was propagated intratesticularly in seronegative New Zealand white rabbits as previously reported [20]. Briefly, three rabbits were injected with 5×10^7^ *T. pallidum* cells per testis and checked daily for disease progression. Animals were euthanized approximately 10 days after infection, at peak orchitis, to recover the highest number of organisms before the onset of immune clearance.

Testes were minced in 20 ml of PBS for approximately 10 min and suspensions were centrifuged twice for 10 minutes at 1,000× G to remove large host cellular debris. The supernate was then centrifuged at 18,000× G for 15 minutes to pellet treponemes. Treponemes were resuspended in 1 ml of PBS and stored on ice as the gradients were prepared. Discontinuous sodium and meglumine diatrizoate (Renografin-60, Bracco Diagnostics, Princeton, NJ) gradients were prepared at room temperature by first diluting Renografin-60 stock solution (60%) to the desired concentrations with PBS [6]. To obtain the discontinuous gradient, a first layer of 60% Renografin-60 (1.5 ml total) was deposited in the bottom of a 10 ml Ultra-Clear Thinwall centrifuge tube (Beckman-Coulter, Fullerton, CA), followed by 1 ml each of 37.5%, 25%, and 19% Renografin-60 dilutions, respectively. Approximately 0.2 ml of ice cold treponemal suspension was carefully layered onto each gradient and tubes were centrifuged at 20°C for 45 min at 100,000× G in an Optima XL-100K ultracentrifuge (Beckman-Coulter) equipped with a SW-41 Ti rotor. Fractions of approximately 0.2 ml were recovered by drop from the bottom of the tube. Fractions containing high numbers of treponemes (identified by dark-field microscopy) were pooled together and treated with a total of 5 units of RQ1 DNaseI (Promega, Madison, WI) to reduce the contamination by rabbit DNA. After treatment and heat inactivation of the enzyme (10 min at 65°C), an appropriate volume of 50× lysis buffer for DNA purification (final concentration: 10 mM Tris, pH 8.0; 0.1 M EDTA; 0.5% w/v sodium dodecyl-sulfate) was added to the treponemal suspension. DNA extraction was performed using the QIAGEN Genomic-tip 100/G kit (Qiagen Inc., Chatsworth, CA), according to the manufacturer's instruction and the sample was stored at −20°C until use.

The list of the additional strains used here can be found in Table 1. Strain propagation, harvest and DNA isolation for amplification and DT-sequencing protocols were performed as previously described [11], [17]. Although we cannot formally evaluate treponemal growth rates for the strains used in this study, differences were found regarding the time between strain passage into the rabbit hosts, and the yield of treponemes at the time of harvest. Some strains were transferred every 10–12 days (Nichols, Chicago, Sea 81-8, SS14, and UW249C), others every 15–30 days (Bal 7, Sea 87-1, Mexico A, Bal 9, Sea 86-1, Sea 81-3, and Bal 3), while some required ≥30 days (Sea 87-1, UW231B, and UW236B). Treponemal yields varied from approximately ≥10^8^ treponemal cells/ml of testicular extract (Nichols, Chicago, SS14), ∼10^7^ cells/ml (Bal 7, Bal 9, Sea 86-1, Sea 81-3, Bal 3, UW231B, and UW249C), and ∼10^6^ cells/ml (Sea 87-1, Mexico A, Sea 81-8, and UW236B).

**Table 1:** *T. pallidum* strains used in this study.

Strain name	Source	Location	Year of isolation	Provided by
Chicago	Primary chancre	Chicago	1951	Paul Hardy and Ellen Nell (Johns Hopkins University, Baltimore, MD)
Nichols (Seattle)^1^	Cerebrospinal fluid	Washington DC	1912	James N. Miller (University of California, Los Angeles)
Nichols (Farmington)^1^	Cerebrospinal fluid	Washington DC	1912	Justin D. Radolf (University of Connecticut Health Center)
Nichols (Dallas)^1^	Cerebrospinal fluid	Washington DC	1912	Michael V. Norgard (University of Texas, Southwestern Medical Center)
Nichols (UCLA)^1^	Cerebrospinal fluid	Washington DC	1912	David Blanco (University of California, Los Angeles)
Nichols (Houston)^1^	Cerebrospinal fluid	Washington DC	1912	Steven J. Norris (University of Texas, Health Sciences Center, Houston)
Mexico A	Primary chancre	Mexico	1953	Paul Hardy and Ellen Nell
Yobs	Lymph node	Atlanta	1965	Paul Hardy and Ellen Nell
Sea81-4	Primary chancre	Seattle	1980	Isolated by Sheila A. Lukehart (University of Washington, Seattle WA)
Sea81-3	Cerebrospinal fluid	Seattle	1981	Isolated by Sheila A. Lukehart (University of Washington, Seattle, WA)
Sea81-8	Cerebrospinal fluid	Seattle	1981	Isolated by Sheila A. Lukehart (University of Washington, Seattle, WA)
Sea86-1	Cerebrospinal fluid	Seattle	1986	Isolated by Sheila A. Lukehart (University of Washington, Seattle WA)
Sea87-1	Cerebrospinal fluid	Seattle	1987	Isolated by Sheila A. Lukehart (University of Washington, Seattle, WA)
Bal-3	Blood	Baltimore	Not known	Paul Hardy and Ellen Nell
Bal-7	Cerebrospinal fluid	Baltimore	1976	Paul Hardy and Ellen Nell
Bal-9	Liver	Baltimore	Not known	Paul Hardy and Ellen Nell
Street Strain 14 (SS14)	Skin	Atlanta	1977	Sexually Transmitted Disease Laboratory Program, Centers for Disease Control, Atlanta (GA)
UW104B	Blood	Seattle	2002	Christina Marra (University of Washington, Seattle, WA)
UW231B	Blood	Seattle	2004	Christina Marra (University of Washington, Seattle, WA)
UW236B	Blood	Seattle	2004	Christina Marra (University of Washington, Seattle, WA)
UW249C	Cerebrospinal fluid	Seattle	2004	Christina Marra (University of Washington, Seattle, WA)

1 Nichols isolates currently propagated at these locations.

### Evaluation of DNA purity

The percentage of rabbit genomic DNA in the Chicago sample was determined by quantitating the copy number of the rabbit (*Oryctolagus cuniculus*) cystic fibrosis conductance transmembrane regulator (RCFTR) gene and the *T. pallidum* TP0574 gene (which encodes for the 47 kDa antigen) by quantitative real-time PCR (qRT-PCR). Primer sequence, amplification protocol and standard curve preparation for the TP0574 gene were previously reported in detail [11]. RCFTR-S (5′-gcgatctgtgagtcgagtctt-3′) and RCFTR-As (5′-cctctggccaggacttattg-3′) primers (Oligos Etc. Inc., Wilsonville, OR) were used to determine the rabbit CFTR gene copy number. Amplification was carried on for 45 cycles in a Roche LightCycler 2.1 instrument (Roche, Basel, Switzerland) using the Master*^plus^* SYBR green kit (Roche) according to the manufacturer's instruction. The reaction conditions for these amplifications included a 10 sec denaturation step at 95°C, an 8 second annealing step at 60°C, and an extension step for 10 sec at 72°C. Acquisition temperature was set at 83°C upon amplicon melting curve analysis. The standard curve for the rabbit CFTR gene was prepared as for the TP0574 gene [11]. The sizes of the rabbit and *T. pallidum* genomes were taken into account to determine the percentage of rabbit DNA in the sample.

### *T. pallidum* DNA preparation and genome sequencing

Genomic DNA isolated from the *T. pallidum* Chicago strain was further processed for Illumina-based sequence analysis using the Paired End DNA Sample Prep Kit (Illumina Inc., San Diego, CA) following the provided protocol. Genome sequencing was performed at the Center for Genome Research and Biocomputing (CGRB) at Oregon State University (Corvallis, OR) using a Genome Analyzer *IIx* System (Illumina Inc.). A first draft of the Chicago strain genome was assembled using the reference-guided assembly program Maq [21] with the *T. pallidum* Nichols strain genome [6] (GenBank accession number for Nichols is NC_000919) as reference. Regions in the reference-guided assembled genome where Maq could not resolve sequence were then compared to contiguous sequences assembled through the use of the *de novo* assembly software VCAKE [22], and a single contiguous draft sequence was then produced. Nucleotide differences between matched pairs were identified using the Diffseq program from the Emboss software suite. The locations and effects of individual differences were first determined using an in-house SNP parsing program (not currently online but available upon contacting the authors) and then re-evaluated after the annotation of the Chicago strain was completed.

### DT-sequencing

Regions containing nucleotide differences between the Chicago and Nichols (Houston) strain were targeted by PCR amplification and conventional DT-sequencing to confirm the high-throughput sequencing data. DNA from both Chicago and the Nichols-Houston strain sequenced in the original *T. pallidum* genome project were used as template. A subset of these regions were selected randomly, and others were selected to confirm differences in genes possibly implicated in generation of diversity in the *tprK* gene (TPChic0897) or transcriptional control (such as TPChic0924, encoding the toxin expression gene, also known as *tex*). Overall, 41 loci (39.8% of the total originally reported [23]) carrying small indels were sequenced in both strains. Twenty six additional regions carrying small indels were amplified using DNA from the Chicago strain and sequenced to further confirm the reliability of the high-throughput sequencing approach.

Primers (designed using the Primer 3 software, http://frodo.wi.mit.edu/primer3/) are in File S1. All PCR amplifications were performed in 100 µl reactions containing 200 µM each dNTP, 20 mM Tris-HCl (pH 8.4), 1.5 mM MgCl_2_, 50 mM KCl, 400 nM of each primer, and 1.0 U of Taq DNA Polymerase (Promega, Madison, WI) with approximately 100 ng of DNA template in each reaction. Cycling conditions were denaturation for 5 min at 95°C, followed by 1 min at 95°C, annealing for 1 min at 60°C and extension for 1 min at 72°C for a total of 45 cycles. A final extension of 10 min at 72°C was included. Amplicons were purified using the QIAgen PCR purification Kit (Qiagen Inc.) according to the provided protocol, and the concentration of each sample was determined using a ND-1000 instrument (NanoDrop Technologies, Wilmington, DE). Sequencing was performed at the Department of Biochemistry DNA Sequencing Facility of the University of Washington, Seattle, WA. Electropherograms were analyzed using the BioEdit software (http://www.mbio.ncsu.edu/BioEdit/bioedit.html).

Amplification and sequencing of 16 ORF fragments found to carry authentic SNPs between the Chicago and Nichols strains were also performed on 12 additional *T. pallidum* strains (SS14, Bal 3, Bal 7, Bal 9, Sea 81-3, Sea 81-8, Sea 86-1, Sea 87-1, Mexico A, UW231B, UW236B, and UW249C). Sequencing of the TP0924 (*tex*) gene region (containing the C→A transversion that truncates the putative Tex protein in Chicago) was also performed using DNA template from various Nichols isolates maintained in different laboratories over the last two decades (Seattle, Farmington, Dallas, and UCLA), as described above.

### *T. pallidum* Chicago strain genome annotation and comparative genome-level analysis

The Chicago strain genome sequence was submitted to the J. Craig Venter Institute (JCVI) Annotation Service (http://www.jcvi.org/cgi-bin/annotation/service/submit/annengine.cgi), where it was processed through JCVI's prokaryotic annotation pipeline. Included in the pipeline are 1) a gene-finding function with Glimmer, HMM, and TMHMM (Hidden Markov Models and Trans Membrane Hidden Markov Models, respectively) searches; 2) frame shift mutation identification through Blast-Extend-Repraze (BER) searches; 3) SignalP predictions for identification of signal peptides; and 4) automatic annotations from AutoAnnotate. The manual annotation tool Artemis (www.sanger.ac.uk/Software/Artemis/v11/) was used to manually review the output from the JCVI Annotation Service and compare it with the Nichols strain genome annotation (Nichols GenBank accession number is NC_000919).

To assess the genome-wide nucleotide diversity of protein-coding genes in Chicago and Nichols genomes, each gene was subject to a modified version of ZPS [24] to perform in batch mode ClustalW-based sequence alignment [25], followed by calculation of the rates of nonsynonymous (dN) and synonymous (dS) mutations using the mutation-fraction method of Nei and Gojobori [26].

## Results

### Comparative gene annotation analysis

Paired-end sequencing yielded a single circular contig devoid of sequence gaps. The Chicago genome [23] was found to be 1,139,281 bp long, in contrast to 1,138,011 bp in the published Nichols genome, suggesting that genomic differences might contribute to explain the differences in infection phenotypes associated with the Nichols and Chicago strains. Next-generation Illumina sequencing was not adversely affected by residual rabbit DNA, corresponding to ∼18% of the total DNA content of the sample, and the coverage of the Chicago genome ranged from ∼50× to ∼100× (average depth coverage was 64×).

Based on the annotation service provided by the JCVI, there were some ORF assignment discrepancies between the Nichols and Chicago genomes that were due to differences in the annotation algorithm rather than any sequence differences. The Chicago genome annotation identified 96 putative ORFs not previously identified in Nichols (File S2). The size of these ORFs was relatively small, ranging from 111 to 399 bp (average length = 180 bp). To facilitate direct ORF comparisons between *T. pallidum* strains, we named the new ORFs based on their proximity to a coding sequence shared by both strains. (For example, according to our nomenclature, TPChic0005a is an ORF annotated only in Chicago and located immediately downstream, either on the plus or minus strand, of TPChic0005 that is homologous to Nichols TP0005. If multiple new ORFs follow a shared annotation, their order is reflected by the alphabetical letter following the ORF. TPChic1025a and TPChic1025b, for instance, follow TPChic1025 and precede TPChic1026. On the other hand, 21 published Nichols ORFs (File S3) were not identified by the JCVI annotation software in the Chicago genome sequence despite nucleotide sequence conservation between the two strains.

Also, the annotation service provided by the JCVI permitted the re-analysis of the possible functions of some *T. pallidum* ORFs shared by two genomes. Among a total of 842 genes with the same annotation, a total of 158 ORFs (File S4) previously listed as hypothetical or conserved hypothetical proteins in the Nichols annotation were now assigned putative identities. Newly annotated possible functions include tyrosine kinases (TPChic0024, TPChic0139), efflux pumps (TPChic0901, TPChic0965, TPChic0988), and permeases (TPChic0301, TPChic0302). New putative lipoproteins (TPChic0069, TPChic0087, TPChic0149, TPChic0625 TPChic0646, TPChic0693), outer membrane lipoprotein carriers and permeases (TPChic0333, TPChic0580, TPChic0582), and metal transporters with outer membrane subunits (TPChic0034, TPChic0035, TPChic0036) were also identified.

Over 99% of all predicted protein-coding genes shared between Chicago and Nichols strains were syntenic (having same relative position in both genomes), thereby arguing against any major role of gene shuffling in shaping the genotypic/phenotypic differences between these two strains. No gene inversions were identified.

### Analysis of single nucleotide polymorphisms

Because the Chicago strain *tprK* is hypervariable with respect to Nichols, a consensus sequence for the seven variable (V) regions of this gene could not be obtained and, thus, are not accounted for in the nucleotide-based comparative analysis. In the complete Chicago genome sequence found in GenBank, the *tprK* V1–V7 region sequences are replaced by N's.

For the Chicago genome, comparison of Illumina sequencing data with traditional DT-sequencing of genomic regions carrying SNPs showed perfect agreement between the two sequencing methods. Although we previously reported [23] that preliminary comparison with the published Nichols genome [6] identified the presence of 44 SNPs between Chicago and Nichols, recent DT-sequencing of the regions carrying these SNPs in the Nichols (Houston) strain, revealed a high frequency of sequencing errors in the published Nichols genome sequence [6]. Overall, only 20 authentic SNPs are found between Chicago and the Nichols genome: four are located within intergenic regions and 16, all non-synonymous, within ORFs coding for putative proteins (Table 2). The SNPs were evenly split between C/T and A/G transitions and were not clustered, but distributed more or less evenly along the genome (File S5).

**Table 2:** Mutations within ORFs and intergenic regions (IGR).

Locus Tagin Chicago	Locus Tagin Nichols^1^	Product	Strand^2^	Amino acid change and position in the ORF Nichols→Chicago	Nucleotide change Nichols→Chicago and genomic position	Length in Chicago^3^ (aa)	Length in Nichols^3^ (aa)
TPChic0051	TP0051	Peptide chain release factor 1, PrfA	F	S (104)→P (104)	T (59887)→C (59897)	351	351
TPChic0076	TP0076	Sugar ABC transporter, permease protein	F	L (198)→V (198)	C (83983)→G (83994)	276	273
TPChic0265	TP0265	Branched-chain amino acid transport system II carrier protein, BrnQ	R	P (230)→L (230)	G (277587)→A (278806)	455	455
TPChic0299	TP0300	Ribose/galactose ABC transporter, ATP-binding protein	F	D (320)→G (389)	A (313326)→G (314550)	585	74
TPChic0430	TP0430	V-type ATP synthase subunit K	F	F (70)→L (70)	T (458815)→C (460043)	140	140
TPChic0433	TP0433	Hypothetical protein	F	V (232)→A (232)	T (461227)→C (462456)	256	256
TPChic0434	TP0434	Hypothetical protein	F	L (31)→V (17)	C (461335)→G (462564)	213	227
TPChic0443	TP0443	Conserved hypothetical protein	F	T (120)→A (103)	A (469810)→G (471039)	267	284
TPChic0488	TP0488	Methyl-accepting chemotaxis protein	F	V (295)→A (422)	T (522138)→C (523372)	718	845
TPChic0584	TP0584	Conserved hypothetical protein	F	A (314)→T (314)	G (634785)→A (636027)	469	469
TPChic0621	TP0621	TprJ protein	R	F (572)→V (567)	A (673514)→C (674758)	755	758
TPChic0746	TP0746	Pyruvate, phosphate dikinase	R	E (501)→G (501)	T (810274)→C (811525)	901	901
TPChic0748	TP0748	Cytoplasmic filament protein A	R	C (31)→Y (31)	C (814853)→A (816100)	678	678
TPChic0790	TP0790	Antibiotic transport protein, putative	R	C (168)→R (139)	A (857221)→G (858469)	859	888
TPChic0924	TP0924	Tex protein	F	Y (529)→STOP	C (1004582)→A (1005844)	545	788
TPChic0978	TP0978	LspA signal peptidase II	F	F (195)→L (184)	T (1062062)→G (1063328)	186	197

Locus Tag in Chicago	Size (bp)	Nucleotide difference Nichols→Chicago	Location in Chicago
IGR 3′ of TPChic0222	268	A→G substitution	228663
IGR 3′ of TPChic0222	268	1 C insertion	228664
IGR 3′ of TPChic0582	47	G→T substitution	635067
IGR 3′ of TPChic0683	292	T→A substitution	749498
IGR 3′ of TPChic0698	102	G→A substitution	767635

1 [6].

2 F indicates the forward or plus (+) strand; R indicates the reverse or minus (−) strand.

3 Diversity in size reflects differences between the annotation of the Chicago genome and the annotation of the Nichols genome [6].

To further explore whether these genomic differences between Nichols and Chicago genomes could have been promoted by the extensive propagation of the Nichols strain in the rabbit host, we analyzed the identity of each ORF-associated mutation in 12 other *T. pallidum* strains (Table 3) which, like Chicago, were propagated in rabbits far less extensively than Nichols. As a result of these 14 genome cross-examinations of 16 SNP regions, we identified only one SNP accumulated in Chicago (in TPChic0746, Table 3). Because the other 12 *T. pallidum* strains were identical to Nichols for this nucleotide position, we define such a change as “Chicago-specific”. Interestingly, the remaining 15 SNPs were determined to be “Nichols-specific”, in that Chicago and the other 12 genomes had identical nucleotides in these polymorphic positions, with the exception of the *tprJ* gene (TpChic0621) where one of the 12 other strains (Bal 7, Table 3) showed a sequence identical to Nichols. These findings clearly demonstrate that 12 other strains analyzed here are significantly more similar to Chicago at the DNA level. Overall, these data strongly suggest that “Nichols-specific” SNPs were acquired through mutation, and not recombination; furthermore, because all the “Nichols-specific” SNPs predict amino acid changes in their respective putative proteins, such a significant predominance of “Nichols-specific” changes suggests functional adaptation of Nichols in the rabbit host. Of the 16 polymorphic genes targeted in our analysis, 12 (75%) genes were annotated with defined functions, equivalent to 729 (74%) total genes with defined functions in the annotated Chicago genome. Although this small set of genes did not permit us to statistically evaluate over-representation of functional categories, at least four of these polymorphic genes are known to encode putative virulence factors, possibly contributing to the phenotypic differences seen during infection between Nichols and Chicago. These genes are TPChic0488 (Methyl-Accepting Chemotaxis protein), TPChic0621 (TprJ protein), TpChic0922 (Tex protein, discussed later in more detail), and TpChic0978 (LspA Signal Peptidase II).

**Table 3:** SNP analysis in *T. pallidum* strains.

Locus Tag in Chicago	Locus Tag in Nichols^1^	Product/Functional Category	Amino acid change and position in the ORF Nichols→Chicago	Chicago, Bal 9, Sea 86-1, Sea 81-3, Mexico A, Sea 81-8, Bal 3, UW231B, UW236B, UW249C, SS14, Sea 87-1	Bal 7	Nichols
TPChic0051	TP0051	Peptide chain release factor 1 (PrfA)/Transcriptional regulator- DNA binding protein	S (104)→P (104)	C	C	N
TPChic0076	TP0076	Sugar ABC transporter, permease protein/Membrane-Transport	L (198)→V (198)	C	C	N
TPChic0265	TP0265	Amino acid ABC transporter, permease protein (BfnQ)/Membrane-Transport	P (230)→L (230)	C	C	N
TPChic0299	TP0300	Ribose, galactose ABC transporter, ATP-binding protein/ATP binding- Transport	D (320)→G (389)	C	C	N
TPChic0430	TP0430	V-type ATPase, subunit K (AtpK-1)/Membrane-Transport	F (70)→L (70)	C	C	N
TPChic0433	TP0433	Hypothetical protein/Uncharacterized	V (232)→A (232)	C	C	N
TPChic0434	TP0434	Hypothetical protein/Uncharacterized	L (31)→V (17)	C	C	N
TPChic0443	TP0443	Conserved hypothetical protein/Uncharacterized	T (120)→A (103)	C	C	N
TPChic0488	TP0488	Methyl-accepting chemotaxis protein (Mcp2-1)/Membrane-Signal transducer	V (295)→A (422)	C	C	N
TPChic0584	TP0584	Conserved hypothetical protein/Uncharacterized	A (314)→T (314)	C	C	N
TPChic0621	TP0621	Tpr protein J/Nucleotide binding	F (572)→V (567)	C	N	N
TPChic0746	TP0746	Pyruvate, phosphate dikinase/ATP binding	E (501)→G (501)	N	N	N
TPChic0748	TP0748	Cytoplasmic filament protein A (CfpA)/Cytoplasmic protein	C (31)→Y (31)	C	C	N
TPChic0790	TP0790	Antibiotic transport protein, putative/Membrane-Transport	C (168)→R (139)	C	C	N
TPChic0924	TP0924	Toxin expression protein (Tex)/RNA binding-Hydrolase activity	Y (529)→STOP	C	C	N
TPChic0978	TP0978	Signal peptidase II (LspA)/Membrane-Proteolysis	F (195)→L (184)	C	C	N

1 [6].

C = identical to Chicago.

N = identical to Nichols.

The most direct way to detect any action of positive selection in protein-coding genes is to evaluate whether the rate of amino acid replacement (dN, nonsynonymous mutation per non-synonymous nucleotide site) is significantly higher than the rate of silent, synonymous mutations (dS, synonymous mutation per synonymous nucleotide site), assuming silent mutations to be, in general, of a neutral nature. Due to the small number of SNPs and because all changes were non-synonymous, the dN/dS rate could not be evaluated directly either for individual genes or for all the polymorphic genes concatenated. If, however, for the sake of analysis we incorporate a synonymous change in the concatenated genes with SNPs, the resulting dN/dS value of 4.5 (0.00081/0.00018) shows that dN was significantly higher (P = 0.03) than dS. Therefore, the absence of any synonymous SNP in the observed dataset strongly indicates that the genetic changes are positively selected and, likely, of an adaptive nature.

### Analysis of insertions and deletions

Apart from a single large event involving a 1204 bp insertion in an intergenic region (position 148519–149723 in the Chicago genome), indel analysis at the time the Chicago genome was released on GenBank [23] identified 103 small (≤3 nt) insertions/deletions between the two genomes (involving a total of 109 nt, due to the presence of 4 di-nucleotide indels, and 1 tri-nucleotide indel). DT-sequencing of 41 loci carrying such indels in both Chicago and Nichols (Houston) revealed however that, with the exception of two loci (TPChic0667, and the IGR 3′ of TPChic0222), the above result was due to sequencing errors in the 1998 Nichols genome. DT-sequencing of “indel-carrying” loci using template DNA from the Chicago strain never showed discrepancies with the Illumina-based sequencing results. A list of erroneous differences (both SNPs and indels) between the Nichols and Chicago strains is reported in File S6. Although only 39.8% of the originally reported differences due to indels were re-analyzed using DT-sequencing in both strains, it is striking that only 2 indels out of 41 (4.8%) were confirmed as real. This indicates that the total extent of differences due to true indels between the Nichols and Chicago strains is likely to be significantly more limited than originally reported.

A single C nucleotide insertion within the TPChic0667 ORF (coordinates: 730194–731009) caused a frame shift and an early termination of the ORF with respect to Nichols' paralogous gene. As a result, when Nichols and Chicago annotations are compared, Nichols' TP0667 ORF (555 codons) encompasses both Chicago's TpChic0667 ORF (275 codons) and TPChic0667a (271 codons). This indel was found to be “Chicago-specific”, based upon analysis by DT-sequencing of the same locus in 12 more *T. pallidum* strains (data not shown). A single C insertion (position: 228663) was also confirmed in the intergenic region 3′ of TpChic0222 (Table 2). Indels that were identified by comparative genomic analysis but are not currently confirmed by DT-sequencing using the Nichols (Houston) strain are reported in Table 4. Indels falling within homopolymeric nucleotide sequences were found in three Chicago ORFs (TPChic0127, TPChic0479, and TPChic0618), and within 3 intergenic regions (3′ of TPChic0026, TPChic0121, TPChic0621).

**Table 4:** Differences within ORFs and intergenic regions (IGR) not confirmed by DT-sequencing in the Nichols strain.

Locus Tag (Chicago)	Locus tag^1^ in Nichols	Product	Strand^2^	Nucleotide difference(s)	ORF coordinates in Chicago	Length in Chicago (aa)	Length in Nichols^1^ (aa)	Confirmed by DT-sequencing in Chicago
TPChic0065	TP0065	Hypothetical protein	F	G insertion	71726:72313	195	195	Yes
TPChic0126	TP0126	Hypothetical protein	R	C insertion	147627:148310	227	291	No
TPChic0127	TP0127	Hypothetical protein	F	GG deletion	149834:150193	119	229	No
TPChic0132	TP0132	Hypothetical protein	R	G insertion	154158:154352	64	69	No
TPChic0135	TP0135	Hypothetical protein	R	C insertion	156755:157243	162	313	Yes
TPChic0177	TP0177	Hypothetical protein	R	G deletion	195519:196931	470	436	No
TPChic0208	TP0208	Preprotein translocase subunit, SecY	F	G insertion	214955:216274	439	450	No
TPChic0217	TP0217	Chaperone protein, DnaJ	F	G insertion	221992:223242	416	324	No
TPChic0248	TP0248	Hypothetical protein	F	C deletion	262114:262587	157	134	No
TPChic0263	TP0236	Hypothetical protein	F	G insertion	275427:277034	535	594	Yes
TPChic0279	TP0279	Bifunctional cytidylate kinase/ribosomal protein S1	F	G deletion	295567:298056	829	863	Yes
TPChic0329	TP0329	Serine hydroxymethyl transferase, GlyA	F	G deletion	351513:353054	5135	574	Yes
TPChic0348	TP0348	Hypothetical protein	R	G insertion	374026:375150	374	469	Yes
TPChic0376	TP0376	Hypothetical protein	R	C insertion	402077:402973	298	301	Yes
TPChic0377	TP0377^3^ TP0378	Flagellar basal body-associated protein, FliL	F	G deletion	403076:403555	513	574	Yes
TPChic0397	TP0397	Flagellar basal-body rod protein, FlgC	F	G deletion	423486:423944	152	129	Yes
TPChic0407	TP0407	Hypothetical protein	F	C deletion	432163:432843	224	226	No
TPChic0415	TP0415	Hypothetical protein	F	G insertion	443468:443950	160	177	Yes
TPChic0424	TP0424	Hypothetical protein	F	G deletion	453081:453695	204	232	Yes
TPChic0425	TP0425	Hypothetical protein	F	GG insertion	453695:454240	181	86	No
TPChic0477	TP0477	Hypothetical protein	R	C insertion	507750:508454	234	241	No
TPChic0479	TP0479	Hypothetical protein	R	C insertion	510135:510692	185	224	Yes
TPChic0486	TP0486	Hypothetical protein	R	T deletion	518618:520213	581	484	Yes
TPChic0491	TP0491	Hypothetical protein	F	G insertion	526253:527308	351	344	No
TPChic0520	TP0520	Hypothetical protein	R	C insertion	562151:562975	274	151	Yes
TPChic0535	TP0535	Conserved hypothetical protein	R	C insertion	579388:579687	99	70	No
TPChic0536	TP0536	Hypothetical protein	R	2 insertions (C, C)	579729:579968	79	133	No
TPChic0555	TP0555	Serine/threonine sodium symporter	R	G insertion	602163:603344	393	396	Yes
TPChic0594	TP0594	Hypothetical protein	R	C deletion	647488:648165	225	202	Yes
TPChic0597	TP0597	Hypothetical protein	R	4 insertions (G, A, A, A)	650054:652117	687	605	No
TPChic0618	TP0618	Hypothetical protein	R	C deletion	671051:671440	129	119	No
TPChic0651	TP0651	Hypothetical protein	R	2 insertions (G, C)	714371:716938	803	855	Yes
TPChic0731	TP0731	Hypothetical protein	F	G insertion	797978:798598	206	236	Yes
TPChic0762	TP0762	Conserved hypothetical protein	F	C insertion	826405:827505	366	393	Yes
TPChic0830	TP0830	Hypothetical protein	F	C insertion	897880:898665	261	280	No
TPChic0856	TP0856	Hypothetical protein	F	C insertion	934375:935559	394	325	No
TPChic0993	TP0993	Hypothetical protein	R	G insertion	1078502:1079527	341	318	No
TPChic0995	TP0995	Cyclic nucleotide binding protein	F	G insertion	1080774:1082108	444	428	No
TPChic0976	TP0976	Conserved hypothetical protein	F	G deletion	1060425:1061900	491	459	No
TPChic1031	TP1031	TprL protein	F	2 insertions (C, G)	1125339:1127168	609	514	No

Locus Tag in Chicago	Size(bp)	Nucleotide difference(s)	Location in Chicago	Possible function	Confirmed by DT^3^ -sequencing
**IGR 3′ of TPChic0026**	178	G triplet insertion	34076	Transcriptional regulation of the *fliG1* gene^2^	No
**IGR 3′ of TPChic0121**	220	1 C insertion	140954	Unknown	No
**IGR 3′ of TPChic0126**	228	1 G insertion	148354	Unknown^2^	No
**IGR 3′ of TPChic0162**	175	1 C insertion	185722	Unknown	Yes
**IGR 3′ of TPChic0233**	134	1 C insertion	243077	Unknown	No
**TPChicr01**	1140	1 G insertion	231516	Unknown	No
**IGR 3′ of TPChic0277**	128	1 C insertion	295223	Unknown	No
**IGR 3′ of TPChic0349**	212	1 C deletion	375777	Unknown	Yes
**IGR 3′ of TPChic0380**	140	1 G insertion	408391	Unknown	Yes
		1 G insertion	408433	Unknown	Yes
**IGR 3′ of TPChic0408**	41	GC insertion	436204	Unknown	Yes
**IGR 3′ of TPChic0598**	37	1 G insertion	652150	Unknown	No
**IGR 3′ of TPChict30**	95	1 C insertion	659642	Unknown	Yes
**IGR 3′ of TPChic0621**	58	1 G deletion	676478	Transcriptional regulation of the *tprJ* 2 gene	No
**IGR 3′ of TPChic0893**	173	1 G deletion	973872	Unknown	Yes

1 [6].

2 F indicates the forward or plus (+) strand; R indicates the reverse or minus (−) strand. *fliG1*, TPChic0126, and *tprJ* are located in the minus strand.

3 The Chicago ORF to the left encompasses these Nichols ORFs.

Among the “Nichols-specific” indels, the only mutation targeting intergenic regions appeared to be the 1204 bp deletion corresponding to the region downstream of TPChic0126 and upstream of TPChic0127 (spanning the location of TPChic0126a/b/c regions in the reverse strand and TPChic0126d in the plus strand). Šmajs *et al.* [27] previously reported that a subpopulation of the Nichols (Houston) strain used in the original *T. pallidum* genome project does not carry such deletion, suggesting that this genomic region might not be stable within a single treponemal isolate. The 1204 bp insertion lies between two direct repeats of 24 bp (aatgtatttcagggtgtctttctc), suggesting a loop-out mechanism for this deletion.

## Discussion

**Figure 1:**
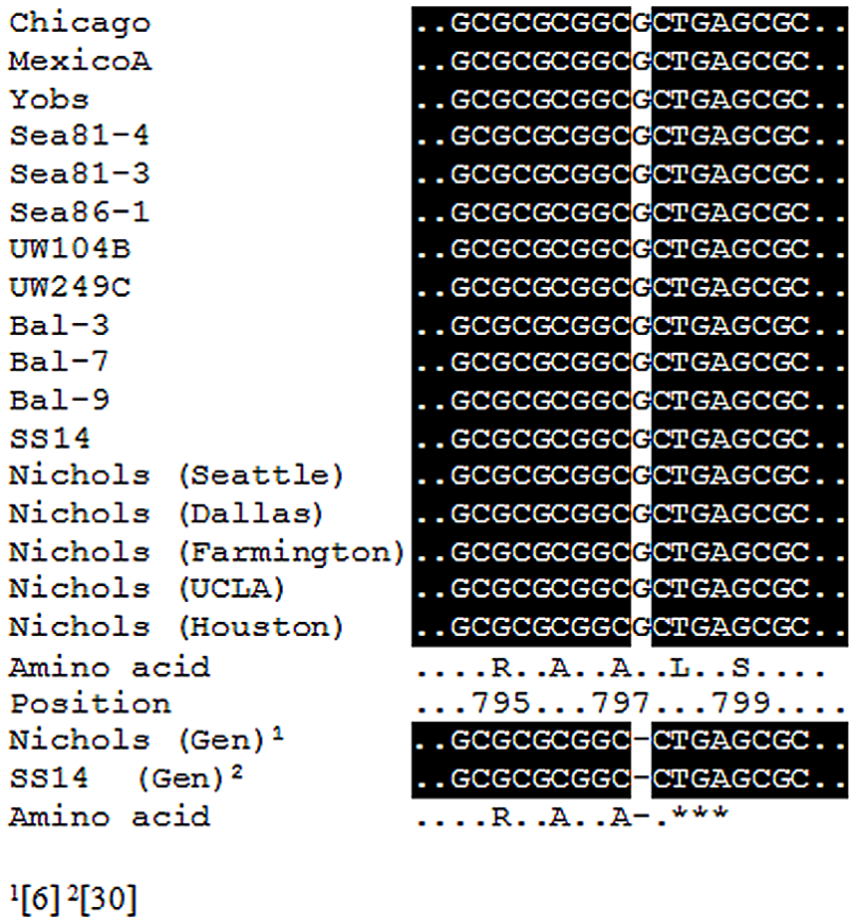
TPChic0899 sequence alignment in *T. pallidum* isolates. The single artifactual G deletion in the Nichols strain results in a frameshift and puts in frame the TGA triplet resulting in a premature stop codon and two ORFs (TP0899 and TP0900) was not confirmed in either the several Nichols lineages (Seattle, Dallas, Houston, UCLA, and Farmington) and in the eleven other non-Nichols *T. pallidum* isolates examined. Amino acid position is indicated according to the Chicago strain genome annotation. Nichols (Gen) and SS14 (Gen) refers to the genome sequences already available for these strains in GenBank (accession numbers are NC_000919 and CP000805.1, respectively).

Chicago and Nichols differ in their origins of isolation (primary chancre vs CSF), durations of propagation in the rabbit host, gene expression levels, induction of antibody and cellular immune responses to some antigens, and rates of TprK variation, the latter being higher in Chicago than in the Seattle Nichols [19]. With respect to the published Nichols genome sequence, a 1204 bp insertion was found in the intergenic region downstream of TPChic0126. This large insertion contains 19 putative donor sequences used by *T. pallidum* to generate variability within all of the seven *tprK* V regions, especially V3 and V6 [19]. Although this insertion might be speculated to be a reason for Chicago's higher *tprK* variability, this 1204 bp fragment is also present in the Nichols strain currently propagated in our laboratory [18], which is slow to develop *tprK* variants. Therefore, the number of donor sites alone cannot explain the relative hypervariability of Chicago *tprK*. The Nichols strain has been extensively propagated in rabbits and this might have selected for a *tprK* sequence that is optimal for survival and rapid growth in rabbit tissues. Frequent passage of the Nichols strain (every 9–12 days) for routine propagation, virtually in the absence of an adaptive immune response, might have permitted the reduction in Nichols' propensity to vary *tprK*. Comparative analysis between the two strains did not show differences in the genes coding for the recombination machinery typically involved in gene conversion (i.e. *ruv* and *rec* genes, genes encoding site-specific recombinases or hypermutation homologues; data not shown). Structural predictions of the TPChic0899 ORF obtained using the Bio Info Bank Metaserver (http://meta.bioinfo.pl) however, found the encoded protein to be similar to an AddB-like deoxyribonuclease, a component of the counterpart of the *E. coli* RecBCD enzyme in Gram positive bacteria. TPChic0899 spans Nichols' TP0899 and TP0900 (originally annotated as separate hypothetical proteins) [6]. The presence of two ORFs in Nichols is due to a single G deletion that puts in frame the TGA triplet introducing a premature stop codon. Because of the possible involvement of this enzyme in homologous recombination, we further explored this difference between Chicago and Nichols. DT-sequencing of the region containing the G insertion was performed in a total of 16 *T. pallidum* isolates, including Nichols strains obtained from several laboratories and the SS14 strain (also reported carrying the deletion; GenBank accession number CP000805.1) [28]. Our sequencing data revealed that the G nucleotide is actually present in all isolates (Figure 1) confirming that the annotation of two separate ORFs, TP0899 and TP0900, in Nichols [6] and SS14 [28] is indeed erroneous. Because this gene appears to be functional in all *T. pallidum* strains, it is, therefore, likely not associated with the increased rates of *tprK* variation that Chicago exhibits with respect to Nichols. Nonetheless, this example underscores the likelihood, when comparative genome-wide studies among *T. pallidum* strains are pursued, of encountering inaccuracies in available sequences.

**Figure 2:**
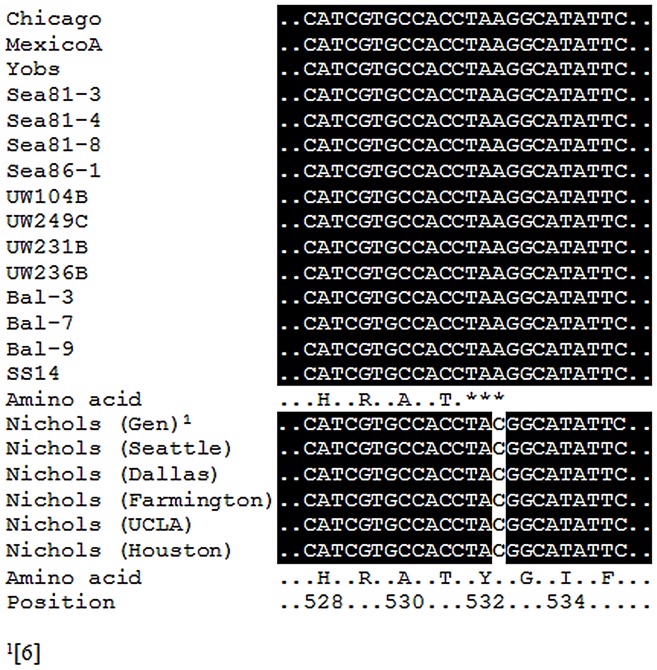
*tex* gene sequence alignment in *T. pallidum* isolates. The C→A transversion that generates a premature TAA (stop) codon in the TPChic0924 gene was found in eleven other non-Nichols *T. pallidum* isolates. No truncating mutation was found in the *tex* gene from the Nichols isolate currently propagated in our laboratory (Nichols Seattle), and in the Nichols strain used in the orginal *T. pallidum* genome project (Houston) [6], or in Nichols strains obtained from other laboratories (Dallas, UCLA, and Farmington). Amino acid position is assigned according to the published Nichols strain genome annotation [6]. Nichols (Gen) refers to the genome sequences already available for this strain in GenBank (accession number is NC_000919).

TPChic0924, which encodes the Tex transcriptional regulator, could potentially explain reported differences in transcription of some *tpr* genes in Chicago vs. Nichols [11]. The Chicago Tex protein is predicted to be 250 aa shorter than in Nichols. Tex was first isolated and characterized in *Bordetella pertussis* by virtue of its negative effect on the transcription and expression of toxin genes *ptx* and *cyaA* [29]. Tex paralogs were then identified in a wide variety of bacterial species [30], [31] and were shown to contain domains involved in nucleic acid binding [31]. Interestingly, studies conducted on the *Pseudomonas aeruginosa* Tex protein showed that presence of the carboxyl-terminal domain (present in Nichols but not in Chicago) permits Tex to bind nucleic acids [31] and thus inhibit transcription. The presence or absence of a complete Tex protein in *T. pallidum* could affect a strain's ability to express virulence factors. To further support the “Nichols-specific” nature of this change, it is found that all examined non-Nichols *T. pallidum* isolates carry the same A/C transversion (Figure 2) that would truncate the Tex protein in Chicago, in sharp contrast with the five Nichols isolates (Seattle, Houston, Dallas, Farmington, and UCLA), where the ORF encoding the Tex protein would not be truncated.

When the Chicago genome was first released [23], we reported that 44 coding sequences, annotated as independent ORFs in Nichols, are fused in Chicago leading to 21 considerably longer genes. TPChic0006, for instance, was predicted to be 417 aa long, and to span Nichols' TP0006-0008 (51, 216, and 89 aa, respectively). It is however evident now that these initial observations were a result of sequencing errors in the original Nichols genome, and not the result, as initially postulated, of gene inactivation of original longer sequences by frame shift or nonsense mutations. Recently, Šmajs and collaborators [32] suggested that genomic decay might have played a central role in *T. paraluiscuniculi*'s adaptation to the rabbit host and loss of infectivity to humans [33], and the hypothesis that gene inactivation in the Nichols strain could reflect its adaptation to rapid passage in rabbits for nearly a century, also appeared plausible. Resequencing of the Nichols (Houston) genomic regions containing mutations hypothetically responsible for inactivation of these genes, however, clearly revealed that these annotation differences are also due to sequencing errors in the Nichols genome. It is therefore very likely that reannotation of the resequenced Nichols genome will be significantly more similar to that currently reported for Chicago. Similar findings were described by Cejková *et al.* [34]. A complete list of predicted gene fusions is reported in File S6.

Indels falling within homopolymeric nucleotide sequences were found in three Chicago ORFs (TPChic0127, TPChic0479, and TPChic0618), and within 3 intergenic regions (3′ of TPChic0026, TPChic0121, TPChic0621). Growing evidence suggests that changes in the length of these homopolymeric repeats, likely induced by slipped-strand mispairing during DNA replication, might be involved in transcriptional or translational control of *T. pallidum* genes. For example, the poly-G repeat upstream of TPChic0621 (TprJ) was shown to control transcription of this gene through a phase variation mechanism that allows transcription only when the poly-G tract is eight (or fewer) nucleotide-long [17]. The poly-G repeat upstream of TPChic0026 (encoding the *fliG1* gene) could have a similar role, although evidence of intra-strain variability of this homopolymeric tract is currently not available. Furthermore, recent evidence suggests that changes in the poly-G repeat within TpChic0127 could either cause a frameshift that prematurely truncates the putative TP0127 protein, or change its reading frame, resulting in a novel protein of approximately equal length but with a different amino acid sequence (unpublished data). Variation in the homopolymeric tracts associated with TPChic0479, and TPChic0618 can also influence the annotation of these ORFs.

Analysis of SNPs in protein-coding genes showed only nonsynonymous mutations, suggesting the presence of recent diversification favoring structural changes in *T. pallidum* genomes. Overall, significantly higher rates of nonsynonymous changes in the Nichols genome indicate positive selection pressures in 16 protein-coding genes throughout the genome. Limited frequency of polymorphic genes did not permit us to determine whether these genes with recent structural changes could be grouped into specific functional categories of proteins. However, we found a strong clustering of polymorphic genes into two functional groups – membrane proteins and DNA-binding proteins. Within the set of genes with defined functions, the single “Chicago-specific” SNP accumulated in an ATP-binding protein-coding gene, while most of “Nichols-specific” SNPs were found to be in membrane protein-coding genes mostly related to transport and proteolysis (Table 3).

Our study suggests that genetic variability likely influences the phenotypic differences seen between the Nichols and Chicago strains of *T. pallidum* [10], [11], [35], even though definitive evidence for the correlation between specific genomic change(s) and phenotypic differences will require further investigation. This study also raises an important concern regarding the selection process that led to these mutations, believed to result from the adaptation of the Nichols strain to the rabbit host. Our comparative analysis incorporating 12 more *T. pallidum* strains for the regions carrying SNP changes in Nichols and Chicago, indeed initially suggested that this might be the case, and that the SNPs identified in Chicago and Nichols might reflect pathoadaptive changes the Nichols strain acquired following years of growth in the laboratory animal where it has been propagated so far. Interestingly however, in the DAL-1 genome (GenBank accession number NC_016844) [34], a *T. pallidum* strain recently isolated from the amniotic fluid of a pregnant woman [36], most of the Chicago/Nichols polymorphic loci were identical to Nichols sequences. Based on this evidence, we cannot exclude that Nichols and DAL-1 represent a separate naturally-occurring clonal lineage within *T. pallidum*. The significant predominance of non-synonymous polymorphisms between Chicago and Nichols strains strongly suggests the likelihood of a role of positive selection in microevolution of *T. pallidum* strains, whether due to differential adaptation during rabbit passage or pathoadaptation of individual strains in the human host.

Support for the mutational evolution of Nichols from an ancestral *T. pallidum* lineage also comes from the published genome of *T. paraluiscuniculi* (Cuniculi A strain, GenBank accession number NC_015715.1), closely related to *T. pallidum* [37]. In the Cuniculi A strain, nine of the Chicago/Nichols polymorphic loci (TP0051, TP0265, TP0430, TP0443, TP0488, TP0584, TP0748, TP0790, and TP0978) are identical to non-Nichols strains that were analyzed here, confirming the “Nichols-specific” nature of the mutations. Ongoing research in our laboratories using comparative genomics on a population-wide scale will provide an insight into phylogenetic relationships of *T. pallidum* clonal populations and likely will help explain the role of such sequence changes during syphilis infection.

## Supporting Information

File S1 **Primers used in this study.**
(PDF)

File S2 **Newly identified ORFs in** ***T. pallidum*** **Chicago strain not described in the previously annotated Nichols genome** [**6**] **.**
(PDF)

File S3 **ORFs in** ***T. pallidum*** **Nichols strain that are not annotated in the Chicago strain genome.**
(PDF)

File S4 **Hypothetical new protein function in** ***T. pallidum*** **Chicago strain genome.**
(PDF)

File S5 **Schematic view of circular maps showing the distribution of mutations in Chicago (inner grey circle) and Nichols (outer black circle) genomes.** The map shows SNPs in protein-coding regions as pink and blue bars according to their location in the forward or reverse strand, respectively. The large deletion of 1204 bp in Nichols was shown as green arrow. The start/end and the scale values along the genomes are denoted in base-pairs (bp).
(TIF)

File S6 **SNPs and indels initially identified by comparative analysis between Chicago and Nichols that were found to be due to sequencing errors in the published Nichols (Houston) genome** [**6**] **.** Table S6.1: changes involving SNPs; Table S6.2: Changes involving indels and SNPs.
(PDF)
