# Supplementary material for: Footprint of Positive Selection in Treponema pallidum subsp. pallidum Genome Sequences Suggests Adaptive Microevolution of the Syphilis Pathogen
Source: PLoS Negl Trop Dis. 2012 Jun 12;6(6):e1698. doi: 10.1371/journal.pntd.0001698 (PMC3373638; doi:10.1371/journal.pntd.0001698)
Supplement: File S1 — Primers used in this study. (PDF) [file pntd.0001698.s001.pdf]

File S1. Primers used in this study

Name[1]	Sequence (5'→3')	Amplicon size (bp)
0006F	GGTTGTGTATGGGGACTCGT	365
0006R	TGCCTGACTAACCACAATGG	
0007F	AAGGAGCAATACGCCAATGT	346
0007R	ACCAATGACGCTCACAACAG	
0013F	GCACGTTTCTTTTCCTCGTC	271
0013R	CATGCCATCTTTGCTTACCA	
0018F	TTTTCTGGCTCGTTGATCACT	368
0018R	GAAAGCGAAATAATCCCAGAAA	
0020F	CTCGTTTCATGATATTGCATCAG	360
0020R	GTCCTTCTGCAAAGCTACCG	
0049F	CTGGTTTTTCTGCCCCAGAT	323
0049R	TGCAGAACTCACCGTCATGT	
0051F	ACAATGGCGCTATGAAGTCC	331
0051R	ACGACAATGCCCGTAGGTAG	
0065F	TTTCTCGTGCGTCATTGTGT	268
0065R	AACAGGGCGCTGTATACCTTT	
0076F	TCACGGATAGTGGTGTCGAA	270
0076R	CCACAGCCCCATACATTTTC	
0082F	TTGCGAACTCGCAGTATTTG	227
0082R	AAACGCGAGGAAAAGGATTC	
0083F	GCCACTGAAGAAATCTCAGACA	360
0083R	CTCAGAAAGAGACAGCGCATC	
0098F	CGTCTGCACTGCTCCAATAA	354
0098R	CGCAGGCGCTTAAGTATCAT	
0106F	TTATTTGACGTGGTTCTTCTCC	365
0106R	GTCTGTTGCAACCCCATTCT	
0135F	AATTTTGGAAGGCTGTGTGG	265
0135R	GGTATTGCACCAGTCCACCT	
0154F	CGTCTAACGTTCCCCACACT	352
0154R	AATACCACCGAAGGCAACAC	
0162igrF[2]	TTGCGTTTTGTTTTCTCCTG	360
0162igrR[2]	GTGCGTAAAACCGGTCAGC	
0172F	AAAGCTCCTCGTCAGAATGG	272
0172R	TGCTCACCCTCACCATATCA	
0174F	GACCTCCTCCGGTGAAAATA	273
0174R	CAGGGCCAAGTCTTGAAAAA	
0175/0176F	TGCCTTCCAGGGTTGTAGAT	436
0175/0176R	GGATATCGCCTTGACACTGG	
0221F	GCGTACGCAGAGCGATAGAG	332

0221R	GTTTCTGGCAGGGGATGTTT	
0222igrF[2]	CGAATCGAAAGAGTTGATAGAGA	363
0222igrR[2]	TTCCTTCAAGAGCAGCGTTT	
0263F	TGAGGAGACGCTCTTTTTCC	366
0263R	CGCAGCAGAAGAAAGTTCAA	
0265F	AGTAGTGCGATGCCTGCTTT	364
0265R	CGACACTCGGTCCCTACCT	
0278/0279F	AAGGTTGTGTAAGGGTCCTGAA	554
0278/0279R	GGTCCATCAAGAGCGATGAT	
0284F	GACGCGCCCTTTATTGATAG	347
0284R	TGTGCGTAATTTTCTGCAGTG	
0286F	GCGTTTCCCCTTCGTTAAGT	347
0286R	ACCGGTCCCGATGGTATATT	
0288F	GTGCGCTGTTGCTTGTATGT	372
0288R	CAGATCCTGCTCCTTTCTCG	
T30F	GCCTCTTCTGTGGACCTTCC	319
T30R	GTGGTGGAGTCGATCATGG	
0299F	TCTGGGGTATGTTCGGTCAC	254
0299R	TGCTCCTTTCCATCCAGAC	
0324F	GGAAGCGTTTCTGGAGGAC	267
0324R	GTTGACTGTAACGGCACCAG	
0329F	AAGCTCAGTCTGCTCGGAAG	263
0329R	GGCCTCACGCAAGAAGACTA	
0348F	GCGGTGAATTACTTTTTGAGC	309
0348R	GGATGGTATACCTTTCAAAGTGG	
0349igrF[2]	GTGTCATCTCGCAGGGTGTA	314
0349igrR[2]	CCAACCGAGCAACAGCAG	
0350F	CTTGCGAATGGACTGGAAAT	313
0350R	CACGAGGAAATACCGCATC	
0376/0377F	CGATACGATTTGGAAAGACG	563
0376/0377R	GATGACACTCACAGGCTGCT	
0379F	GCTGCTCTTCCATGTGAACC	360
0379R	ACGCACTGGGAGGTGAGG	
0380igrF[2]	GGAGCGTGCACATCAAGTAA	405
0380igrR[2]	AACGCACTCTTTTGGAATCG	
0379F	ACGCACTGGGAGGTGAGG	340
0379R	GCTGCTCTTCCATGTGAACC	
0397F	TGTGGATGCTGAGCAAGAAG	286
0397R	TCTTCTGCGCCAAAACTACC	
0408igrF[2]	TCAGGATCCAGCAAACAAAA	372
0408igrR[2]	AGACATACGACGCCGATAGC	
0415F	GGGTAGGGATAGGACGGAAG	348

0415R	CACTTCGGTGGCTGATGTTA	
0419F	CGTTGGCTGCACTTTCCT	300
0419R	AAGCCTGGAACGCAAAATC	
0424F	GGCAGGGGTAGAGTTGAGGT	273
0424R	AGAAACGTAGCGCACAATCC	
0430F	GTTCGTGGAAGCGCTGTTAT	312
0430R	CAAAAACCATCACCAGAAGC	
0433F	ATGTTCCCCAAAAACACTGC	365
0433R	TCACGAGACAAAGGGGAAAC	
0434F	TGAGGTCGCTTCTCAGCATA	642
0434R	CAGAAAGGCGTCCTTGAATC	
0443F	CGCGTTGGTAGATGACATGA	528
0443R	CGCCTGGAGCTCTACAAAAG	
0462F	GCAGTGTGCTTTGTTTTTGG	313
0462R	GCGGATGCTCGGTATTTTTA	
0463F	CCACTGTCGTACGGTCCAAG	340
0463R	AACGGTGTAAATGTCGCTGA	
0468F	GTGCACACGTCCAAGCTCTA	474
0468R	TACACCCGTATGGGAGAAGC	
0479F	ACAAAAGAACGTCGGTCCTG	270
0479R	GGAGGGCTTGACACTCTCAC	
0481F	CAGGCAGATGGAGGAAAGAA	359
0481R	ACGTCCAGGAGGAACGAAAT	
0485/0486F	CCGTGCTGATAATACCGATG	526
0485/0486R	GGATGCCCAGCACTTGTTAT	
0487F	GCAAAAAGAGCGACACAAGA	952
0487R	AGCGTTTCTGACGATGGACT	
0488F	AATTATATCCGCCTGGCAAA	341
0488R	CCGTACGCGATGTCTTCTAA	
0520F	TATTGAGCCGTTCGATTTCC	339
0520R	CACGCGCAGAATTCATTGTA	
0533F	TTGGAGAAAAATCCCGACAC	486
0533R	GTTTTTCGCTCATGGTACGC	
0555F	CGAGACGAGACGGAGCTATC	326
0555R	GGTGCTTGATGTGTTGCACT	
0567F	TCCTGTAGCTCAGCGTCCTT	348
0567R	GGTGGCTCAAACGTTGTCTT	
0575F	AAGGCAATTACCCACGTGAC	301
0575R	CACCTTCGGTTACCAAACCA	
0582igrF	AACTCTTTTTGCGCACGAGT	432
0582igrR	GGGAAGTTTCTGGCAACTCA	
0584F	CTCCGCGAGTTTGAAGAATC	301

0584R	GCTCAACGTTTACGCATTCC	
0587F	GCGTCTGCGAACTTTACTCC	460
0587R	CGCATCTTGTGCAGGTAGAA	
0587F	AGCGTTGTGCAGTACCTGTG	305
0587R	ATCCTTGCAGGACTCGCTTA	
0591F	TAGGGCAAGAAGAAGGCTCA	310
0591R	GATCTGAAAGAGCGCGATTC	
0594F	TTCGGGTGAGTAAGGAGGAA	319
0594R	AGATCCTGCAAGCGGTAAGT	
0597F	TGCGCACAATGAAGAAACTC	414
0597R	TGTGTGGATAGAGCCTGCTG	
0621F	GGTCACCGCTCTTAGTTTCG	428
0621R	GCAGAACAGCATTCTGACCA	
0633F	GGCTCCGTTACTGCATCAAT	430
0633R	GTAACCAAAGGACGGGTGAA	
0640F	CAGCAGTCTGCCACTGAGAC	343
0640R	AAAGAGCAGTCCCCTCTTCC	
0667F	GATTACCTTCCGCGTCACAC	348
0667R	CGCCACAGTTTGTGGTAATG	
0651F	CCTTCTTCCTGCGAGCATAC	430
0651R	GGTCTGTCGCACTCCTTCTC	
0683igrF	TGAGCAGCCGGGTTTGGACG	222
0683igrR	CGCAACTATCACTCCTCTCG	
0698igrF	GTGCAGGCAGAAGTACTTGA	208
0698igrR	TACCCACACAACCCCCACTC	
0702F	GTCCTTCCCCCAGTGGTACT	328
0702R	GATGCTTGAAGCGCAGGT	
0731F	GTGTACGCCTGAGGTACGTG	316
0731R	GGGGAGTAGGACCGATGACT	
0746F	AAGATCATACGGCGGAACAC	351
0746R	GATCTCTTTGGTCCGTCCAG	
0748F	AAGCACCCACATCAACCTTC	432
0748R	AGCAGGAGGTGAACCAGCTA	
0762F	CGCTTTGGATACGCAACACT	328
0762R	CACTGAAGGGAACACAGAAA	
0781/0782F	GACGATAGGTTCCCTGCGTA	394
0781/0782R	GCGTTAGGAAATGCGTTGAT	
0790F	CCTCATTCCCCATTTCGTTA	327
0790R	TCCTGTCTGCCGAATACCTT	
0805F	CTCCGCCTCACCTTCTACAG	357
0805R	GGAAGTGCACAGACAAACGA	
0813F	GCATACCAGCGTGTAGCTGA	339

0813R	ACGGCACCCTCAGATAGTGT	
0854F	GGGACGTTAGTGCTGAAAGC	334
0854R	GCTCCTCCTGATTACGTTGC	
0859F	ACGTATCGCACGCTTTCTCT	431
0859R	CCCCACATTGTAGAGCCACT	
0865F	AAGGCAGACATCCCAATACG	339
0865R	AGCGCGTACAATTGCAGATA	
0866R	TTTGGGAACAACGCCATAAT	301
0866F	ACATCGTGCCAAAGGAAAAT	
0893igrF[2]	GGGGCTCACTCTAGCATGTTT	370
0893igrR[2]	GTGATCGGTGGGGTGTTATC	
0899F	GCCCCGGTCTTGTAATCTAA	360
0899R	TGTAGTACTCGAGCGGCTGT	
0924F	TTGGGGTGGGACTCTATCAG	348
0924R	CAAAAATTATCTTTCCCGTTGC	
0926F	GTACAGCAACTGAGCGTTCC	320
0926R	GAAACCCCACGAGGAAAAA	
0928F	CCGATCAGTACCTTTACCACCT	354
0928R	TCCGATAGGAGTGGGTCATT	
0949.2F	CACGAAGGCAAAAGATGAGG	490
0949.2R	TTTCTTCGCGTTGTTTCACC	
0978F	TGGGTGCTTATGTGCCTACA	389
0978R	CAAGAGCAAACCACAGGTCA	
0983F	GTGGCGGTTCTCTTATACGC	378
0983R	AGGCGGGGATTGTCTTTAAC	
1029.1F	CTCTGCGCACTGAGAATTGC	370
1029.1R	AATCATTGGTGGTGCGAAAC	

[1]Primer names reflect the Chicago strain annotation.
[2]"igr" indicates primers that target intergenic regions.
