# Supplementary material for: Footprint of Positive Selection in Treponema pallidum subsp. pallidum Genome Sequences Suggests Adaptive Microevolution of the Syphilis Pathogen
Source: PLoS Negl Trop Dis. 2012 Jun 12;6(6):e1698. doi: 10.1371/journal.pntd.0001698 (PMC3373638; doi:10.1371/journal.pntd.0001698)
Supplement: File S2 — Newly identified ORFs in T. pallidum Chicago strain not described in the previously annotated Nichols genome [6] . (PDF) [file pntd.0001698.s002.pdf]

File S2. New hypothetical ORFs in Chicago

Locus Tag	Product	Strand	ORF coordinates (left-right)	Length in Chicago (aa)
TPChic0005a	Putative ABC antibiotics transporter	F	6832:7074	80
TPChic0009a[1]	TprA	F	9839:9985	48
TPChic0042a	Hypothetical protein	F	50456:50569	37
TPChic0054a	Hypothetical protein	F	63955:64128	57
TPChic0060a	Conserved hypothetical protein	F	69092:69220	42
TPChic0063a	Iron-sulfur cluster-binding protein	F	71177:71317	46
TPChic0071a	Conserved hypothetical protein	F	78557:78694	45
TPChic0073a	Hypothetical protein	F	80608:80739	43
TPChic0074a	Conserved hypothetical protein	F	82344:82460	38
TPChic0082a	Hypothetical protein	F	92226:92354	42
TPChic0093a	Hypothetical protein reverse	R	102722:102850	42
TPChic0112a	Hypothetical protein	R	129809:129958	49
TPChic0126a	Hypothetical protein	R	148352:148576	74
TPChic0126b	Hypothetical protein	R	148825:149124	99
TPChic0126c	Hypothetical protein	R	149087:149224	45
TPChic0126d	Conserved hypothetical protein	F	149449:149586	45
TPChic0127a	Hypothetical protein	F	150285:150500	71
TPChic0128a	Hypothetical protein	F	151263:151601	112
TPChic0134a	Hypothetical protein	R	156755:157243	162
TPChic0134b	Hypothetical protein	F	157199:157597	132
TPChic0134c	Hypothetical protein	F	157633:158157	174
TPChic0140a	Pyrazinamidase/nicotinamidase	R	162967:163137	56
TPChic0143a	Urea amidohydrolase subunit beta	F	165766:165927	53
TPChic0159a	Hypothetical protein	R	182511:182627	38
TPChic0163a	GroES, 10 kDa chaperonin	F	186793:186951	52
TPChic0179a	Conserved hypothetical protein	F	199684:199839	51
TPChic0206a	RpmD, ribosomal protein L30	F	214289:214486	65
TPChic0217a	Hypothetical protein	R	223274:223402	42
TPChic0240a	Conserved domain protein	F	246634:246765	43
TPChic0248a	Para-aminobenzoate synthase, component II	F	262550:262669	39
TPChic0258a	Hypothetical protein	F	271274:271423	49
TPChic0283a	Conserved hypothetical protein	F	299717:299857	46

TPChic0315a	Hypothetical protein	R	332364:332489	41
TPChic0328a	Hypothetical protein	F	351400:351516	38
TPChic0339a	Hypothetical protein	F	362269:362415	48
TPChic0353a	Conserved hypothetical protein	F	379025:379177	50
TPChic0354a	Hypothetical protein	R	379867:379980	37
TPChic0361a	Conserved hypothetical protein	F	385016:385132	38
TPChic0366a	Putative secreted protein	F	390531:390731	66
TPChic0379a	Hypothetical protein	F	406398:406529	43
TPChic0405a	Putative ectonucleoside triphosphate diphosphohydrolase 6	F	430838:431080	80
TPChic0408a	Membrane protein	F	436311:436688	125
TPChic0420a	Hypothetical protein	F	448945:449067	40
TPChic0437a	Hypothetical protein	R	465399:465515	38
TPChic0437b	Hypothetical protein	R	466269:466385	38
TPChic0466a	Hypothetical protein	F	496292:496405	37
TPChic0474a	Conserved hypothetical protein	F	504468:504617	49
TPChic0487a	Hypothetical protein	F	522097:522222	41
TPChic0524a	Putative Pvstp1 protein	R	568567:568713	48
TPChic0524b	Galactose-specific lectin	R	569229:569381	50
TPChic0536a	Hypothetical protein	R	580063:580188	41
TPChic0538a	Hypothetical protein	R	582512:582631	39
TPChic0547a	Hypothetical protein	F	592734:592946	70
TPChic0574a	Hypothetical protein	R	624814:624987	57
TPChic0575a	hypothetical protein	R	626931:627059	42
TPChic0591a	Conserved hypothetical protein	R	643696:643851	51
TPChic0591b	Hypothetical protein	R	643853:643975	40
TPChic0609a	Conserved hypothetical protein	R	662544:662657	37
TPChic0629a	Hypothetical protein	F	689032:689145	37
TPChic0638a	Hypothetical protein	R	698569:698952	127
TPChic0707a	Hypothetical protein	R	776377:776508	43
TPChic0750a	6- phosphogluconate DH, NAD-binding protein	R	817155:817289	44
TPChic0765a	Conserved hypothetical protein	R	831653:831841	62
TPChic0783a	Hypothetical protein	R	851849:852046	65
TPChic0814a	Hypothetical protein	F	884959:885102	47
TPChic0816a	Conserved hypothetical protein	R	886591:886812	73
TPChic0820a	Hypothetical protein	R	889945:890070	41

TPChic0823a	Hypothetical protein	R	892634:892807	57
TPChic0832a	Hypothetical protein	R	901310:901423	37
TPChic0836a	Hypothetical protein	F	907272:907415	47
TPChic0856a	Conserved hypothetical protein	F	935585:935731	48
TPChic0865a	Glycosyl transferase, group 1	R	945795:946019	74
TPChic0865b	Hypothetical protein	R	946061:946177	38
TPChic0866a	Conserved hypothetical protein	R	948050:948217	55
TPChic0866b	Conserved hypothetical protein	R	948214:948393	59
TPChic0908a	Cytochrome c oxidase subunit 1	R	989856:990104	82
TPChic0913a	Conserved hypothetical protein	F	993128:993310	60
TPChic0919a	Putative lipoprotein	F	997746:997904	52
TPChic0921a	Conserved hypothetical protein	F	1001916:1002968	350
TPChic0924a[2]	Tex protein	R	1006566:1006730	54
TPChic0936a	Hypothetical protein	R	1019980:1020099	39
TPChic0949a	Undefined protein	R	1033037:1033321	94
TPChic0949b	Undefined protein	R	1033325:1033726	133
TPChic0954a	Conserved hypothetical protein	F	1037989:1038282	97
TPChic0971a	Hypothetical protein	R	1056034:1056156	40
TPChic0972a	Hypothetical protein	R	1057517:1057651	44
TPChic0980a	Hypothetical protein	F	1065282:1065401	39
TPChic1002a	Hypothetical protein	R	1092611:1092736	41
TPChic1017a	BLUF domain protein	R	1111067:1111195	42
TPChic1025a	Hypothetical protein	R	1119178:1119465	95
TPChic1025b	Protochlorophyllide oxidoreductase	R	1119472:1119654	60
TPChic1029a	Hypothetical protein	R	1125114:1125227	37
TPChic1036a	Hypothetical protein	F	1135069:1135215	48

[1]TPChic009a corresponds to the TprA protein NH_2-terminal domain. In both Nichols and Chicago the *tprA* gene has a premature stop codon that truncates this ORF, therefore the NH_2- terminal region is recognized as an independent coding sequence by the JCVI annotation.

[2]TPChic924a corresponds to the Tex protein COOH-terminal domain. In Chicago the gene encoding for the *tex* gene has a premature stop codon that truncates this ORF, therefore the COOH- terminal region is recognized as an independent coding sequence by the JCVI annotation.
