# Supplementary material for: Footprint of Positive Selection in Treponema pallidum subsp. pallidum Genome Sequences Suggests Adaptive Microevolution of the Syphilis Pathogen
Source: PLoS Negl Trop Dis. 2012 Jun 12;6(6):e1698. doi: 10.1371/journal.pntd.0001698 (PMC3373638; doi:10.1371/journal.pntd.0001698)
Supplement: File S3 — ORFs in T. pallidum Nichols strain that are not annotated in the Chicago strain genome. (PDF) [file pntd.0001698.s003.pdf]

File S3. Hypothetical ORFs in Nichos that are not annotated in Chicago

Locus Tag in Nichols[1]	Product	Size (aa)	Locus Tag in Nichols	Product	Size (aa)
TP0012	Hypothetical protein	60	TP0573	Hypothetical protein	30
TP0059	Hypothetical protein	75	TP0583	Hypothetical protein	41
TP0129	Hypothetical protein	158	TP0590	ribosomal protein L36	38
TP0130	Hypothetical protein	139	TP0645	Hypothetical protein	58
TP0137	Hypothetical protein	45	TP0656	Hypothetical protein	33
TP0161	Hypothetical protein	30	TP0699	Hypothetical protein	36
TP0169	Hypothetical protein	32	TP0749	Hypothetical protein	73
TP0180	Hypothetical protein	52	TP0759	Hypothetical protein	39
TP0202	Ribosomal protein S14	61	TP0791	Hypothetical protein	81
TP0209	Ribosomal protein L36	37	TP0818	Hypothetical protein	50
TP0224	Hypothetical protein	34	TP0857	Hypothetical protein	106
TP0232	Hypothetical protein	38	TP0867	Hypothetical protein	61
TP0250	Hypothetical protein	75	TP0871	Hypothetical protein	53
TP0311	Hypothetical protein	47	TP0904	Hypothetical protein	83
TP0318	Hypothetical protein	59	TP0909	Ribosomal protein L19	123
TP0332	Hypothetical protein	41	TP0916	Hypothetical protein	42
TP0355	Hypothetical protein	127	TP0932	Hypothetical protein	30
TP0409	Hypothetical protein	61	TP0950	Hypothetical protein	94
TP0467	Hypothetical protein	82	TP0955	Hypothetical protein	90
TP0495	Hypothetical protein	49	TP1030	Hypothetical protein	165
TP0539	Hypothetical protein	73			

[1] [6]
