# Supplementary material for: Footprint of Positive Selection in Treponema pallidum subsp. pallidum Genome Sequences Suggests Adaptive Microevolution of the Syphilis Pathogen
Source: PLoS Negl Trop Dis. 2012 Jun 12;6(6):e1698. doi: 10.1371/journal.pntd.0001698 (PMC3373638; doi:10.1371/journal.pntd.0001698)
Supplement: File S4 — Hypothetical new protein function in T. pallidum Chicago strain genome. (PDF) [file pntd.0001698.s004.pdf]

File S4. Hypothetical new protein function

Locus Tag (Chicago)	Product	Possible function or cellular process	Locus Tag (Chicago)	Product	Possible function or cellular process
TPChic0017	Tetratricopeptide repeat (TPR) domain protein	Unknown	TPChic0502	Putative ankyrin repeat-containing protein	Regulation of antioxidation metabolism
TPChic0024	TrkA domain protein	Tyrosine kinase	TPChic0512	Bifunctional enzyme IspD/IspF	MEP cytidylyltransferase/ MECDP-synthase
TPChic0025	M16 peptidase family protein	Metalloprotease	TPChic0515	Organic solvent tolerance protein	Sugar transporter of phosphotransferase system
TPChic0032	rRNA small subunit methyltransferase	Methyltransferase	TPChic0518	Thiamine pyrophosphokinase	Thiamine pyrophosphate synthesis
TPChic0034	Mzt family protein	Manganese/zinc/iron ABC transporter, substrate-binding protein	TPChic0522	CvpA family protein	Colicin V production
TPChic0035	ATP-binding protein	Metal transport	TPChic0544	Endonuclease/ exonuclease/ phosphatase family	Intracellular signaling
TPChic0036	Membrane protein	Metal transport	TPChic0559	ThiI protein	Thiamine biosynthesis/tRNA modification protein
TPChic0042	Putative LysM domain protein	Glucan-binding protein/ glucosyltransferase	TPChic0563	DnaJ domain-containing protein	Chaperone
TPChic0046	Tpl protein	Signal peptidase II-like activity	TPChic0564	Putative membrane protein	Unknown
TPChic0050	Phosphoribosyl transferase domain protein	Purine salvage	TPChic0572	Putative FMN-binding domain protein	Unknown
TPChic0052	PrmC protein	Methyltransferase	TPChic0580	Lipoprotein releasing system	Permease
TPChic0054	TrmH family protein	RNA methyltransferase	TPChic0582	Lipoprotein releasing system	Permease
TPChic0055	Sodium pump decarboxylases, gamma subunit	Na+ transport across membranes	TPChic0587	HolA protein	DNA polymerase III, delta subunit
TPChic0067	Tetratricopeptide repeat (TPR) domain protein	Unknown	TPChic0601	Dxr protein	1-deoxy-D-xylulose 5-phosphate reductoisomerase
TPChic0068	Cfr family protein	Generation of catalytic radicals	TPChic0603	UppS protein	Di-trans,poly-cis-decaprenyl cistransferase
TPChic0069	Putative lipoprotein	Unknown	TPChic0612	SufB protein	FeS assembly

TPChic0073	HD domain protein	Metal-dependent phosphohydrolases	TPChic0613	SufD protein	FeS assembly
TPChic0079	Putative aldehyde oxidase and xanthine dehydrogenase family protein	Oxidative metabolism of purines	TPChic0622	Tetratricopeptide repeat (TPR) domain protein	Unknown
TPChic0081	Putative FAD-binding protein	Unknown	TPChic0624	OmpA family protein	Porin
TPChic0086	PilZ protein	Type IV pilus assembly protein	TPChic0625	Putative lipoprotein	Unknown
TPChic0087	Putative lipoprotein	Unknown	TPChic0628	Putative nicotinate phosphoribosyl transferase	Unknown
TPChic0095	Tetratricopeptide repeat (TPR) domain protein	Unknown	TPChic0636	RecO protein	DNA repair
TPChic0120	MetN protein	Methionine import ATP-binding protein	TPChic0646	Putative lipoprotein	Unknown
TPChic0121	Putative radical SAM domain protein	Generation of catalytic radicals	TPChic0648	Tetratricopeptide repeat (TPR) domain protein	Unknown
TPChic0123	Tetratricopeptide repeat (TPR) domain protein	Unknown	TPChic0650	Putative metalloprotease	Unknown
TPChic0139	TrkA domain protein	Tyrosine kinase	TPChic0668	Putative yggt family	Repeat found in conserved hypothetical integral membrane proteins
TPChic0148	Putative membrane protein	Unknown	TPChic0674	Putative Smr domain-containing protein	Endonuclease
TPChic0149	Putative lipoprotein	Unknown	TPChic0691	ScpA/B protein	Segregation and condensation protein
TPChic0153	PAP2 family protein	Type 2 phosphatidic acid phosphatase	TPChic0693	Putative lipoprotein	Unknown
TPChic0154	Putative S4 domain protein	Voltage-gated proton channels	TPChic0702	M23/M37 domain-containing protein	Peptidase
TPChic0155	Putative M23 peptidase domain protein	Beta-lytic metallopeptidase	TPChic0700	M23/M37 domain-containing protein	Peptidase
TPChic0156	Thioesterase family protein	Non ribosomal protein synthesis	TPChic0710	Putative polymerase	Unknown
TPChic0158	HAD-superfamily hydrolase	Haloacid dehalogenase-like hydrolase	TPChic0719	Putative FliO protein	Flagellum biosynthesis

TPChic0181	Putative septum formation initiator subfamily	Unknown	TPChic0730	CDP superfamily protein	Alcohol phosphatidyl-transferase
TPChic0182	Putative YrdC domain	rRNA maturation factor	TPChic0740	HD domain-containing protein	Metal-dependent phosphohydrolases
TPChic0231	RNA pseudouridine synthase superfamily	Formation of pseudouridine in 16S rRNA	TPChic0741	NadD protein	Nicotinate (nicotinamide) nucleotide adenylyltransferase
TPChic0256	Putative von Willebrand factor type A domain	Unknown	TPChic0754	MiaB protein	tRNA-I(6)A37 thiotransferase
TPChic0259	Putative LysM domain protein	Glucan-binding proteins and glucosyltransferases	TPChic0764	HD domain-containing protein	Metal-dependent phosphohydrolases
TPChic0260	Putative lipoprotein	Unknown	TPChic0771	Na+/inorganic phosphate (Pi) cotransporter family protein	Ion transport
TPChic0268	Tetratricopeptide repeat (TPR) domain protein	Unknown	TPChic0796	ApbE lipoprotein	Thiamine biosynthesis
TPChic0282	Tetratricopeptide repeat (TPR) domain protein	Unknown	TPChic0802	DHH superfamily protein	Phosphoesterase
TPChic0288	Cytidylyltransferase domain protein	Aminophosphonate metabolism.	TPChic0815	GNAT family protein	Acetyltransferase
TPChic0290	HAD-superfamily hydrolase, subfamily IIB	Haloacid dehalogenase-like hydrolase	TPChic0819	RnZ protein	Ribonuclease
TPChic0291	FMN-dependent dehydrogenase superfamily	Hydroxyacid oxidase	TPChic0820	Tetratricopeptide repeat (TPR) domain protein	Unknown
TPChic0296	Dephospho-CoA kinase	Pantothenate and CoA biosynthesis.	TPChic0822	Mechanosensitive ion channel family protein	Response to stretching of the cell membrane and to membrane depolarization
TPChic0301	Putative amino acid or sugar ABC transport systems	Permease protein	TPChic0839	Putative Lipoprotein	Unknown
TPChic0302	putative amino acid or sugar ABC transport systems	Permease protein	TPChic0840	Putative transporter, major facilitator family	Unknown
TPChic0307	Pasta domain-containing protein	Penicillin-binding protein	TPChic0854	HAMP domain-containing protein	Cell signaling
TPChic0312	Putative membrane protein	Unknown	TPChic0864	LysM/M23/M37 domain-containing protein	Peptidase
TPChic0333	LolA protein	Outer membrane lipoprotein carrier	TPChic0875	ATP-binding protein	Unknown
TPChic0335	CAAX amino protease	Amino terminal protease	TPChic0876	YeaZ protein	Protease

	family	
TPChic0358	Glycosyl hydrolase	Synthesis and breakage of glycosidic bonds.
TPChic0371	IspE protein	4-(cytidine 5'-diphospho)-2-C-methyl-D-erythritol kinase
TPChic0373	tRNA(Ile)-lysidine synthase	
TPChic0381	Integral membrane protein	Unknown
TPChic0383	MraZ protein	Cell-wall biosynthesis and cell division
TPChic0384	MraW protein	S-adenosyl-methyltransferase
TPChic0385	FtsL protein	Cell division
TPChic0392	Tetratricopeptide repeat (TPR) domain protein	Unknown
TPChic0419	SurE protein	5'-nucleotidase
TPChic0421	Tetratricopeptide repeat (TPR) domain protein	Unknown
TPChic0431	Type III pantothenate kinase	Pantothenate and CoA biosynthesis.
TPChic0436	DHH superfamily protein, subfamily 1	Phosphoesterase
TPChic0438	RdgB/HAM1 family protein	RdgB non-canonical purine NTP pyrophosphatase
TPChic0441	Probable inorganic polyphosphate/ATP-NAD kinase	Phosphorylation of NAD
TPChic0444	Putative LysM domain protein	Glucan-binding proteins and glucosyltransferases
TPChic0449	Putative lipoprotein	Unknown
TPChic0456	Putative lipoprotein	Unknown
TPChic0458	ScpB protein	Segregation and condensation protein
TPChic0459	RNA pseudouridine synthase superfamily	Formation of pseudouridine in 16S rRNA
TPChic0877	HD domain-containing protein	Metal-dependent phosphohydrolases
TPChic0883	YjgP/YjgQ family protein	Permease
TPChic0884	YjgP/YjgQ family protein	Permease
TPChic0901	MATE family protein	Efflux pump
TPChic0906	KH domain-containing protein	RNA processing
TPChic0907	RimM protein	16S rRNA processing protein
TPChic0911	FlhB domain protein	
TPChic0912	HD domain-containing protein	Metal-dependent phosphohydrolases
TPChic0915	Tetratricopeptide repeat (TPR) domain protein	Unknown
TPChic0920	Tetratricopeptide repeat (TPR) domain protein	Unknown
TPChic0937	Phosphodiesterase family protein	Unknown
TPChic0944	Tetratricopeptide repeat (TPR) domain protein	Unknown
TPChic0949	Inner membrane protein OxaA	Integration of membrane proteins
TPChic0954	Tetratricopeptide repeat (TPR) domain protein	Unknown
TPChic0962	Putative efflux ABC transporter	Permease
TPChic0965	RND family protein, MFP subunit	Efflux transporter
TPChic0972	FTR1 family protein	Iron permease
TPChic0975	Tetrapyrrole methylase family protein	Unknown
TPChic0979	TatD family protein	Hydrolase

TPChic0460	Tetratricopeptide repeat (TPR) domain protein	Unknown	TPChic0986	DUF6 protein	Putative integral membrane protein
TPChic0464	TrmB protein	tRNA (guanine-N(7)-)-methyltransferase	TPChic0988	Multiple antibiotic resistance protein	Efflux pump
TPChic0465	Putative lipoprotein	Unknown	TPChic0990	Tetratricopeptide repeat (TPR) domain protein	Unknown
TPChic0470	Tetratricopeptide repeat (TPR) domain protein	Unknown	TPChic0994	TatD family protein	Hydrolase
TPChic0471	Tetratricopeptide repeat (TPR) domain protein	Unknown	TPChic1003	Putative membrane protein	Unknown
TPChic0473	Putative membrane protein	Unknown	TPChic1018	2',3'-cyclic-nucleotide 2'-phosphodiesterase	Purine and pyrimidine metabolism
TPChic0474	Similar to protein Ppro_2673	Unknown	TPChic1029	DbpA protein	RNA-binding domain-containing protein
TPChic0489	Metallo-beta-lactamase family protein	Cell wall synthesis	TPChic1033	Patatin family protein	Phospholipase
TPChic0496	Tetratricopeptide repeat (TPR) domain protein	Unknown	TPChic1034	putative K+-dependent Na+/Ca+ exchanger homolog	Ion transport
