# Supplementary figures and images for: Footprint of Positive Selection in Treponema pallidum subsp. pallidum Genome Sequences Suggests Adaptive Microevolution of the Syphilis Pathogen
Source: PLoS Negl Trop Dis. 2012 Jun 12;6(6):e1698. doi: 10.1371/journal.pntd.0001698 (PMC3373638; doi:10.1371/journal.pntd.0001698)

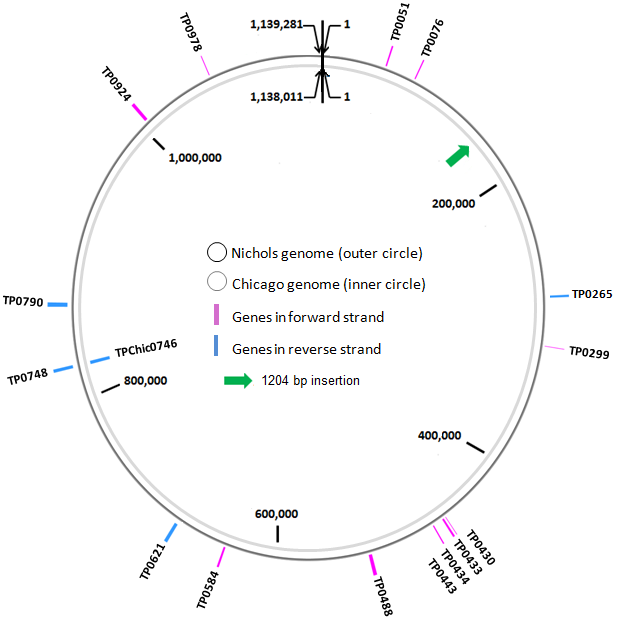

Supplement: File S5 — Schematic view of circular maps showing the distribution of mutations in Chicago (inner grey circle) and Nichols (outer black circle) genomes. The map shows SNPs in protein-coding regions as pink and blue bars according to their location in the forward or reverse strand, respectively. The large deletion of 1204 bp in Nichols was shown as green arrow. The start/end and the scale values along the genomes are denoted in base-pairs (bp). (TIF) [file pntd.0001698.s005.tif]
