# Supplementary material for: Footprint of Positive Selection in Treponema pallidum subsp. pallidum Genome Sequences Suggests Adaptive Microevolution of the Syphilis Pathogen
Source: PLoS Negl Trop Dis. 2012 Jun 12;6(6):e1698. doi: 10.1371/journal.pntd.0001698 (PMC3373638; doi:10.1371/journal.pntd.0001698)
Supplement: File S6 — SNPs and indels initially identified by comparative analysis between Chicago and Nichols that were found to be due to sequencing errors in the published Nichols (Houston) genome [6] . Table S6.1: changes involving SNPs; Table S6.2: Changes involving indels and SNPs. (PDF) [file pntd.0001698.s006.pdf]

File S6.

Changes involving SNPs

Locus Tag in Chicago	Locus Tag in Nichols[1]	Product	Strand[2]	Amino acid mutation(s) and position in the ORF Nichols→Chicago	ORF coordinates in Chicago	Length in Chicago[3] (aa)	Length in Nichols[3] (aa)
**TPChic0020**	TP0020	Hypothetical protein	F	T(154)→S(142)	22088:24175	695	707
**TPChic0106**	TP0106	Choline/carnitine/betaine transporter family protein	R	F(388)→L(388)<br>V(389)→L(389)	118845:120377	510	510
**TPChic0154**	TP0154	Putative S4 domain	F	R(50)→A(109)<br>V(51)→L(110)	177126:178340	404	345
**TPChic0350**	TP0350	Glutamate-5-semialdehyde dehydrogenase, ProA	F	P(154)→A(154)	375863:377149	428	428
**TPChic0379**	TP0379	Preprotein translocase, SecA subunit	R	I(805)→S(805)[4]	403630:406380	916	916
**TPChic0433**	TP0433	Hypothetical protein	F	R(252)→G(252)	403630:406380	256	256
**TPChic0591**	TP0591	HPr(Ser) kinase/phosphatase, HprK	R	F(45)→L(55)[4]<br>V(46)→F(56)	642259:643248	329	319
**TPChic0633**	TP0633	Peptide methionine sulfoxide reductase MsrB/MsrA	F	Y(68)→D(68)	692633:693508	291	291
**TPChic0640**	TP0640	Methyl-accepting chemotaxis protein	F	S(610)→R(610)	700957:702801	614	614
**TPChic0805**	TP0805	Hypothetical protein	R	R(330)→A(326)[4]	874908:876986	692	759
**TPChic0854**	TP0854	Hamp domain-containing protein	R	G(192)→D(192)	926238:930839	1533	1533
**TPChic0983**	TP0983	Conserved hypothetical protein	F	P(69)→R(13)	1067333:1067881	182	238

[1][6].
[2]F indicates the forward or plus (+) strand; R indicates the reverse or minus (-) strand.
[3]Size diversity reflects differences between the annotation of the Chicago genome and the annotation of the Nichols genome [#348].
[4]This change is caused by two SNPs in the same codon.

Changes involving indels and SNPs

Locus Tag in Chicago	Locus Tag in Nichols[1]	Product	Strand[2]	Amino acid mutation(s) and position in the ORF Nichols→Chicago	ORF coordinates in Chicago	Length in Chicago[3] (aa)	Length in Nichols[3] (aa)
TPChic0006	TP0006[4]<br>TP0007<br>TP0008	Putative conserved protein	F	C→T substitution[5]<br>1 C insertion	7008:8261	417	55
TPChic0013	TP0013[4]<br>TP0014	Conserved hypothetical protein	F	GG insertion	12546:13778	410	221
TPChic0018	TP0018[4]<br>TP0019	Hypothetical protein	F	3 insertions<br>(G, G, A)	19371:22052	893	657
TPChic0049	TP0049	Hypothetical protein	R	C insertion and<br>A→G substitution[5]	57507:58598	363	342
TPChic0076	TP0076	Sugar ABC transporter, permease protein	F	G insertion	83403:84233	276	273
TPChic0082	TP0082	Hypothetical protein	R	G insertion	90641:92227	528	600
TPChic0083	TP0083	Hypothetical protein	F	C insertion<br>C insertion	92390:94372	660	426
TPChic0098	TP0098	Hypothetical protein	R	G deletion	106727:107383	218	216
TPChic0172	TP0172[4]<br>TP0173	Hypothetical protein	R	C insertion<br>G deletion	192630:193994	454	175
TPChic0174	TP0174[4]<br>TP0175<br>TP0176	Hypothetical protein	R	G insertion<br>2 deletions (G, G)	194002:195522	506	280
TPChic0221	TP0221	D,D-carboxypeptidase, putative	F	C insertion	227381:228178	260	265
TPChic0284	TP0284[4]<br>TP0285	Conserved hypothetical protein	F	A insertion	299836:301479	547	219
TPChic0286	TP0286[4]<br>TP0287	Conserved hypothetical protein	F	G insertion	301430:302407	325	121
TPChic0288	TP0288[4]<br>TP0289	Cytidylyltransferase domain protein	F	G deletion	302407:304980	857	315
TPChic0299	TP0299[4]<br>TP0300	Ribose/galactose ABC transporter, ATP-binding protein	F	C insertion	313385:315142	585	74
TPChic0324	TP0324[4]<br>TP0325	Conserved hypothetical protein	F	G insertion	341119:345519	1466	485
TPChic0419	TP0419[4]<br>TP0420	5'-nucleotidase SurE	F	T deletion	448160:448930	256	187
TPChic0434	TP0434	Hypothetical protein	F	C→G substitution[7]	448160:448930	256	187

TPChic0462	TP0462[4]<br>TP0463	Conserved hypothetical protein	F	G insertion<br>G deletion	492174:493448	424	280
TPChic0468	TP0468[4]<br>TP0469	Hypothetical protein	R	A→G, T→C, G→A substitutions<br>G deletion	496439:498391	650	176
TPChic0481	TP0481[4]<br>TP0482	Hypothetical protein	F	C insertion	511361:513691	776	477
TPChic0487	TP0487	Hypothetical protein	R	C insertion	520302:522080	592	525
TPChic0533	TP0533	Hypothetical protein	R	G insertion	576681:578366	561	454
TPChic0575	TP0575[6]	Phosphoenolpyruvate-protein phosphotransferase, PtsP	F	C insertion	624908:626980	690	215
TPChic0587	TP0587[4]<br>TP0588	Hypothetical protein	R	G insertion	640838:641860	340	254
TPChic0594	TP0594	Hypothetical protein	R	C deletion	647488:648165	225	202
TPChic0702	TP0702[4]<br>TP0703	M23/M37 peptidase domain-containing protein	R	C insertion	768588:769820	410	226
TPChic0781	TP0781<br>TP0782	Conserved hypothetical protein	R	A deletion<br>G insertion	849653:850603	316	153
TPChic0813	TP0813	Hypothetical protein	R	C insertion	882695:883717	340	428
TPChic0859	TP0859[4]<br>TP0860	Hypothetical protein	F	G deletion	937023:938453	476	232
TPChic0866	TP0866[6]	Putative Mg chelatase homolog	R	C insertion	946144:948084	646	318
TPChic0899	TP0899[4]<br>TP0900	Deoxyribonuclease	R	C insertion	980801:983761	986	797
TPChic0926	TP0926	Signal peptidase I, putative	R	G insertion	1007553:1009388	611	512
TPChic0928	TP0928[4]<br>TP0929	Conserved hypothetical protein	F	G insertion	1009869:1011392	507	246

[1] [6].
[2] F indicates the forward or plus (+) strand; R indicates the reverse or minus (-) strand.
[3] Size diversity reflects differences between the annotation of the Chicago genome and the annotation of the Nichols genome [6].
[4] The Chicago ORF to the left encompasses these Nichols ORFs.
[5] Nucleotide sequence in Nichols→Nucleotide sequence in Chicago
[6] These ORFs contain an authentic frameshift mutation in Nichols not present in Chicago.
[7] This is not the same substitution reported in Table 2.
